# Audition and balance: The impact of echolocation on postural control

**DOI:** 10.1371/journal.pone.0330943

**Published:** 2025-12-08

**Authors:** Chiraz Chouk, Léo Dross, Nicolas Termoz

**Affiliations:** Gipsa-Lab, Move team, UMR CNRS, Saint-Martin-D’Hères, France; CHU Caen: Centre Hospitalier Universitaire de Caen, FRANCE

## Abstract

The links between the perception of acoustic signals and postural control remain largely unexplored, and the control models proposed to date have yet to assign these sensory inputs a role alongside visual, proprioceptive, and vestibular information. Research on this topic has produced varied conclusions, likely due to significant methodological differences. Our study makes novel contributions by aiming to determine the impact of the auditory system—specifically through the perception of echoes—on postural control during quiet standing and to identify the potential link between auditory perception and postural stability. To create controlled acoustic conditions, we developed an automated device representing a reflective object, positioned statically in two locations and dynamically moved in two directions relative to the participants. Eleven naive, blindfolded adult participants (mean age: 23 years ±2.2; mean height: 174 cm ±7.4) with normal hearing underwent kinematic (optoelectronic cameras) and kinetic (force plate) analyses in a semi-anechoic room. After each trial, participants reported their perception of the object’s presence, position, movement direction, and distance. Results demonstrated improved object perception and greater stability in dynamic conditions, particularly when the target moved towards the participants, compared to static conditions. However, no significant correlation between perception and postural stability was observed. Our findings suggest that acoustic information, especially through echo perception, could plays a role in postural control processes alongside visual, proprioceptive, and vestibular input. However, more controlled studies are necessary to examine the relationship between perception and postural stability.

## Introduction

Postural control, the intricate process of maintaining an upright and balanced stance, is a multisensory symphony involving a harmonious interplay of various sensory inputs. To date, different sensory modalities are involved, including visual, vestibular (inner ear), and somatosensory (proprioceptive and tactile) systems [1–4]. In the recent years, the question has been increasingly investigated to what extent auditory information on the afferent side also contributes to balance regulation, and whether the auditory organ should even be considered as fourth pillar of postural regulation. Conventionally, there has been a lack of consensus regarding the role of audition in postural control. A recent review [5] suggested a potential interaction between the auditory and vestibular functions due to their anatomical and physiological proximity. As components of the inner ear, the hearing system, consisting of the cochlea, and the vestibular system, consisting of the utricle, saccule, and the three semicircular canals, are anatomically closely connected, further supporting the hypothesis of functional interaction. This concept remains under discussion, as highlighted by a recent case [6]. The report describes a patient who, after undergoing a neurectomy to the left vestibular nerve, experienced improved balance upon activation of her left sided cochlear implant. During stability assessments on a stable force plate with closed eyes, the patient showed clinically significant enhancements in stability percentage, sway area, and total path length when the cochlear implant was activated. Notably, this improvement occurred despite the patient’s prior left vestibular nerve neurectomy, indicating a significant role of auditory input in balance regulation.

In this framework, it was hypothesized that individuals experiencing hearing loss may face greater difficulty in maintaining balance due to the inability to fully perceive environmental sounds, regardless of their peripheral vestibular function [7]. A similar conclusion was reached by multiple studies [8–10] which observed decrease in postural sway and improvement of gait parameters across various groups. These groups included individuals with normal hearing, both with or without vestibular loss, as well as patients with cochlear implants or hearing aids. In each case, the provision of the auditory input was associated with enhanced balance and stability. While the majority of studies have reported a stabilizing effect of auditory stimuli on balance, some research has found either no effect or even a destabilizing influence [11,12]. These discrepancies are largely due to methodological differences, particularly in two key areas: The first is the choice of postural variables analyzed in the studies. Most biomechanical, behavioral, and physiological studies on posture have focused on the center of pressure (CoP)[13], point of application of the resultant ground reaction forces. However, these studies often overlook the analysis of the center of mass (CoM). Using an inverted pendulum model, these variables are described as the controller (CoP) and the controlled (CoM) elements of the system [14,15]. The CoP constantly oscillates around the vertical projection of the CoM to regulate its position. The preference for focusing on CoP is due to both theoretical and technical reasons—CoP is easier to obtain, making it the most commonly used parameter in studies. However, CoP does not fully account for the dynamics of the CoM. A more relevant, yet more challenging, variable to obtain is the CoP-CoM differential. This variable measures the positional difference between CoP and CoM at each instant and is particularly important because it represents an error signal, closely correlated with the acceleration of the CoM [14–17]. Those variables have been used with both healthy and pathological subjects, type 2 diabetes [18] and blind people [19].

Secondly, we must consider the types of acoustic information involved in this process. While most studies examining the relationship between auditory perception and posture focus on direct sound sources, this link was explored by gradually reducing acoustic cues—transitioning from a live laboratory setting to a soundproof and then a semi-anechoic environment, with white noise diffusion [19]. By comparing sighted and blind individuals, as well as different age groups, his research highlighted the impact of auditory information on postural control. However, these studies often overlook the significant role of indirect acoustic information: echoes. This type of information is extensively utilized by visually impaired individuals to navigate their environments through sound reflection, a technique known as “echolocation.” This technique is traditionally associated with bats and toothed whales [20], but it is also accessible to humans [21]. To echolocate a person emits a sound (a mouth click, Cane tapping...), and then uses sound reflections to obtain information about the environment [22]. In this way, echolocation is an active process that requires both the production of the emission as well as the sensory processing of the resultant sound [23]. People can use echolocation to determine distance, direction, size, material, motion or shape of distal ‘silent’ surfaces (for reviews see [24,25]). After training, normally sighted people are also able to use echolocation to perceive objects, and can develop abilities comparable to, but typically somewhat poorer than, those of blind people [26]. Interestingly, blind individuals can convert received sound signals into spatial images within the visual cortex [27–29]. The brain’s ability to integrate diverse sensory inputs to create cohesive perceptions of the environment showcases its remarkable capacity to exceed the capabilities of individual senses alone [30]. Appreciating the general usefulness of echo-acoustic cues for people, rehabilitation programs were founded based on this technique; it is known by the name of orientation and mobility rehabilitation programs. These programs aim to develop compensatory strategies to enhance autonomy without relying on sensory substitution devices [31]. This intricate relationship between auditory perception and spatial awareness underscores the complexity of human sensory integration and its potential impact on postural control. This ongoing research aims to deepen our understanding of sensory perception, spatial awareness, and motor control, potentially leading to innovative strategies for assisting individuals with visual impairments.

To our knowledge, this study is the first to explore the impact of echolocation on postural control using the most relevant variable for its analysis: CoP-CoM difference. The primary objective is to examine how echoes influence postural stability. Additionally, we aim to identify the relationship between an individual’s ability to perceive objects through echolocation and the observable variations on posture regulation. We hypothesize that participants will be able to perceive the presence of the reflective object and that the acoustic conditions will have a noticeable impact on posture, most likely resulting in a stabilizing effect.

## Materials and methods

### Participants

Eleven young, naive adults (8 men, 3 women; 20–26 years, mean age 23 ± 2.2 years, height 174 ± 7.4 cm) were recruited. Inclusion required adults aged 18–45 years with normal hearing (≤20 dB loss at 125–8000 Hz) confirmed by audiometric testing. Exclusion criteria included neurological or orthopedic disorders and recent musculoskeletal injuries. The recruitment of participants took place between April 15 and April 19, 2024. The study protocol was approved by the Ethics Committee for Research in Science and Technology of Physical and Sports Activities (CERSTAPS: IRB00012476-2024-12-04-307). Potential participants were informed about study procedures and risks. Those who agreed to participate signed an informed written consent form before enrollment.

### Study design and setup

**Fig 1:**
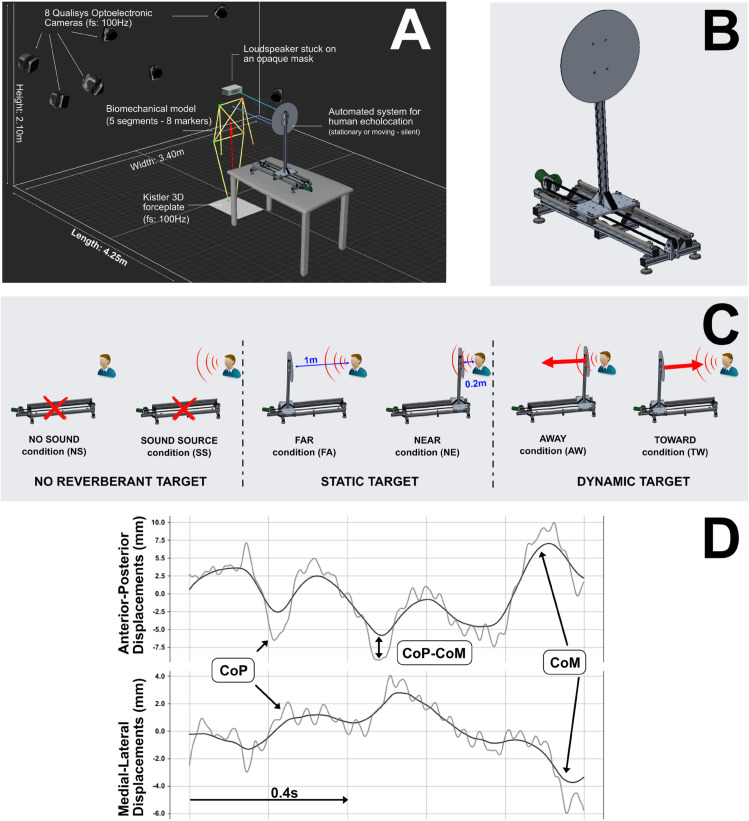
A: Setup in the semi-anechoic chamber in experimental condition. B: Automated device. C: Conditions of the experimental protocol. D: Center of Pressure (CoP) and Center of Mass (CoM) displacements (mm) over time (s).

This study employed a within-subject design to investigate the impact of auditory echoes on postural control during quiet standing. Conducted under controlled laboratory conditions, the experimental plan involved randomized acoustic conditions and repeated measures to assess postural and perceptual responses. The study took place in a semi-anechoic room (3.40m (width), 4.25m (length), 2.10m (height)) (Fig 1A) at University Grenoble Alpes.

The center of pressure (CoP) during the stance task was measured with a 600 mm x 400 mm force plate (Type 9286AA; Kistler Instrumente AG, Winterthur, Switzerland) with a sampling frequency of 100 HZ. In order to obtain the Center of Mass (CoM) of each participant, we used 8 optoelectronic cameras (4 Oqus 600+ and 4 Arqus A12) to modelise the human body with 8 sensors (12.5mm), placed on the internal malleolus, the greater trochanter, the acromion and the styloid process, on each side of the body. Prince et al. [32] reported that a 6-marker model is sufficient to obtain a good estimate of the body center of mass. By using the software Qualisys (Qualisys Track Manager (QTM) : 2021.2) and a python program, we were able to modelise the body in 3 dimensions and get the CoM from anthropometric data.

To accurately simulate diverse acoustic environments and maintain precise control over the conditions, we developed a specialized automated device based on that used in the study by Tirado et al., [33]. This device, designed to represent a reflective target, was strategically positioned in the semi-anechoic room at the participant’s head level, directly facing them.

The device (Fig 1B) consists of a circular target, made of an aluminium disk with a 0.5m diameter and 0.4cm thick, which can move across a 0.80m long rail . The disk was mounted on an adjustable height stand mounted on a platform. A limited sound motor drove the horizontal movement through a cog belt that was mounted between the supporting beams at each end of the rails. A Visaton loudspeaker (50 mm, 90 x 30 mm, 3W) emitting synthesized white noise (frequency range of 20 Hz–20 kHz) was used as stimuli.

### Intervention

Participants were instructed to stood barefoot at the center of a force plate, with feet shoulder width apart and arms relaxed at sides. To eliminate visual cues, participants were blindfolded with opaque Mindfold masks. The mask was topped by a Visaton loudspeaker (50 mm, 90 x 30 mm, 3W). Participants were recuired to wear earplugs during the interval between each trials of static and dynamic conditions to avoid gaining auditory information about obstacle placement.

To accurately simulate diverse acoustic environments and maintain precise control over the conditions, the reflective target, was strategically positioned in the semi-anechoic room at the participant’s head level, directly facing them. Six acoustic environment conditions were established and randomized, with a 20-second duration and 5-minute rest period between each, four trials were conducted for each condition. Participants were instructed to attentively focus on the provided acoustic cues. No specific guidance was provided regarding body posture, except to maintain an undisturbed, quiet, upright standing position with feet placed on a footrest without lifting them. The description of the conditions is provided below: conditions were differentiated to assess the participant’s perception and posture in different reverberant environments (Fig 1C).

- In the “No Reverberant Target” conditions, the participant stood facing away from the automated reverberant device. Two randomized sessions were conducted: (NS) No Sound where no sound source was emitted, and (SS) Sound Source where a sound source was provided without reverberant reflections.
- In the “Static Target” conditions, an automated device positioned a fixed reverberant target in front of the participant at either 0.2m or 1m, with a continuous white noise stimulus present. Two initial feedback trials were conducted, the participant received feedback on the actual target position along with the sound emission, followed by eight trials with the target either at 0.2m (NE) or 1m (FA) from the participant. After each trial, the participant verbally reported the perceived target distance.
- In the “Dynamic Target” conditions, the reverberant target moved at a constant speed (0.05 m.s^−1^) either toward or away from the participant, with the extreme positions fixed at 0.2m and 1m. This also involved 10 randomized 20-second trials: two initial feedback trials where the participant received feedback on the actual target moving direction along with the sound emission, followed by eight trials with the target moving either toward (TW) or away (AW) from the participant. After each trial, the participant reported the perceived direction of target motion.

**Fig 2:**
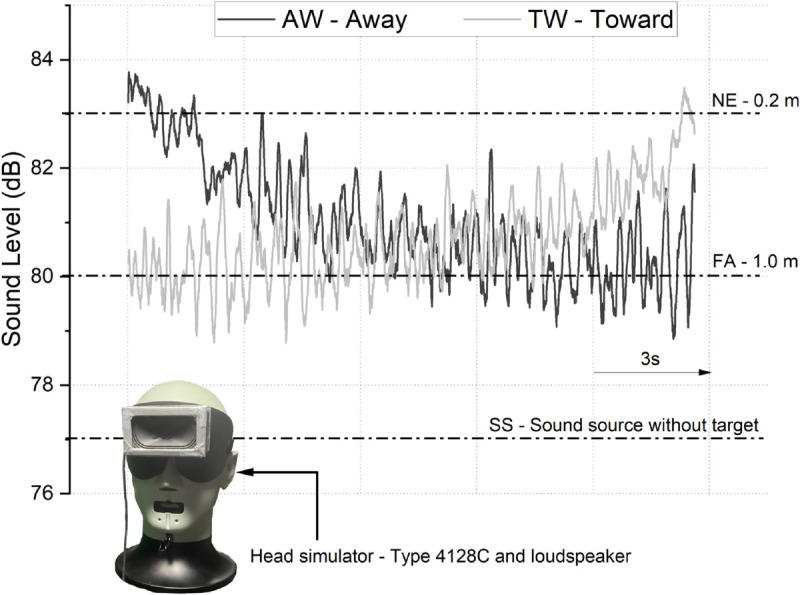
Head simulator (Type 4128C HBK) equipped with the mask and loudspeaker. Sound levels (dB) over time for SS, NE, FA, AW and TW conditions.

We used an instrumented head (head simulator, Type 4128C HBK) equipped with the mask and loudspeaker to measure the sound level reaching the ears for each condition (Fig 2). The following levels were observed: 77 dB for the SS condition, 80 dB for the FA condition, and 83 dB for the NE condition. For the AW condition, the sound level decreased from 83 dB to 80 dB, and conversely, for the TW condition, it increased from 80 dB to 83 dB.

### Data treatment

In our study we chose to analyze the CoP and the CoM kinematics. Postural stability was evaluated by calculating the difference between CoP and CoM (CoP-CoM). The Root Mean square (RMS) was calculated in both A/P and M/L. In our study, the CoP was sampled at 100 Hz, the first and last 5 s of each trial were removed to eliminate any potential startle response the participants might have had to the stimulus onset. Filtering was performed using a dual-pass, second-order, low-pass Butterworth filter with a cutoff frequency of 8 Hz. A model of 8 sets of markers was used to locate the center of mass (CoM) for each participant. From the data provided by the Qualisys sensors, we use the segments of each limb of the human body. The model we used provided 8 points of the human body, which we used to calculate the total-body CoM from anthropometric table.

The COM data were low pass filtered at 8 Hz using a dual-pass second-order Butterworth digital filter. Each individual trial was quality checked by the experimenter, to ensure all markers were labeled correctly by the motion capture system. This was determined by visual inspection of each trial. Data was exported and analysis in Python (3.10).

### Statistic analysis

To analyze the impact of the explanatory variable acoustic condition on the binary response variable perception from repeated measures, we employed a logistic regression with random effects. If acoustic condition was significant after model selection, multiple comparisons controlling the family-wise error rate were performed using Hothorn et al.’s method with the multcomp package in R. To analyze the influence of an explanatory variable (acoustic condition or perception on the response variables RMS in the A /P and M /L axes from repeated measures data. An initial model with fixed effects of the explanatory variable and a random effect of participant was fitted. Likelihood ratio tests were then used to select the appropriate random effects structure, model the variance-covariance matrix, and test the significance of the explanatory variable. P value was fixed to 0.05. The final model was validated by checking residual assumptions, identifying overly influential participants via Cook’s distance, and calculating a pseudo R-squared. If the explanatory variable was significant, multiple comparisons with controlled family-wise error rate are performed using Hothorn et al.’s method.

## Results

### The impact of acoustic condition on the perception variable

**Fig 3:**
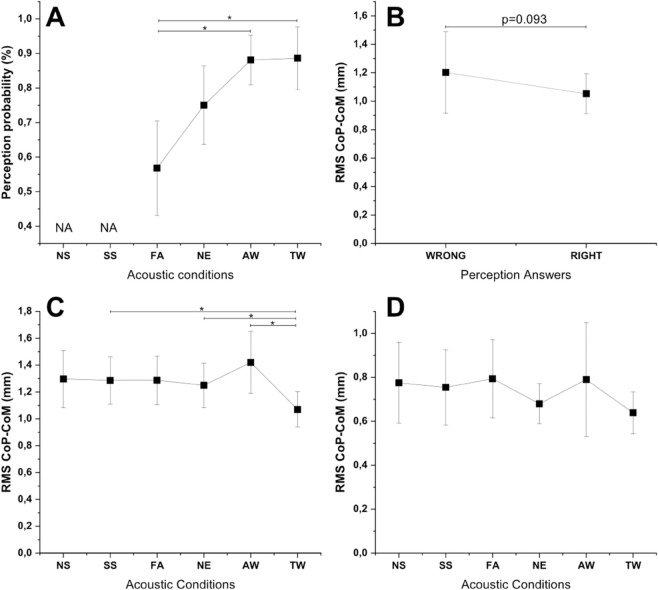
A: Perception probability for each acoustic conditions. B: RMS CoP-CoM for perception answers right or wrong. C: RMS CoP-CoM for each acoustic conditions for the A/P axis. D: RMS CoP-CoM for each acoustic conditions for the M/L axis.

The acoustic condition factor had a significant impact on the perception variable (Chisq (3) = 17.9, p < 0.0001). The results of the multiple comparisons revealed significant differences in the probability of accurate perception between specific conditions (Fig 3A).

### The impact of acoustic conditions on the CoP-CoM postural variable RMS on A/P and M/L axis

On the A/P axis (Fig 3C) : The acoustic condition factor has a significant impact on the RMS (A/P) variable chisq(5) = 21.8, p < 0.0001). The pseudo R-squared value is 0.41, suggesting a moderate proportion of variance explained by the conditions. By a Multible comparaisons, Significant differences (p < 0.05) were observed between the following pairs. On the M/L axis (Fig 3D) : The acoustic condition factor does not have a significant impact on the RMS (M/L) variable (chisq(5) = 7.7, p = 0.16). The pseudo R-squared value is 0.27, indicating a relatively low proportion of variance explained by the acoustic-conditions.

### The link between perception variable and CoP-CoM postural RMS variable under the TW condition

Despite lower RMS CoP-CoM values observed when the subjects answered correctly, the statistical analyses did not reveal significant differences between the perception factor and the RMS variable (chisq(1)= 0.007,p=0. 0.93) (Fig 3B). The pseudo R-squared value is 0.84, indicating a relatively high proportion of variance explained.

## Discussion

The purpose of this study was to exam the impact of the auditory system, through the perception of echoes on the postural control in a quiet standing posture and to identify the potential link between auditory perception and postural control. Examining the influence of various acoustic conditions on target perception revealed a significant impact on the perception. The results indicated a notably higher probability of accurate perception under the TW and AW conditions, where the target was moving, compared to the NE and FA conditions, where the target was stationary. This effect was especially pronounced in the FA condition, with the target fixed one meter away from the participant. These findings underscore the importance of considering acoustic conditions, particularly target motion, in perception studies. The results suggest that target motion may enhance perception, possibly due to changes in acoustic cues or increased attention to the moving target.

Our study measure auditory perception thresholds using a moving target presented via an automated system. This approach aligns with previous research on dynamic acoustic conditions, which suggests that as a listener moves toward a wall, they perceive a low-frequency sound that rises in pitch [31]. This rising pitch when approaching a reflective surface is a compelling auditory phenomenon. Our acoustical measurements clearly show that the amplitude of the reflected white noise decreases with distance. Notably, the amplitude of the reflected noise is greater at close distances and increases over time as it moves forward from the participant. A study by Shinn-Cunningham et al, [34] investigated the acoustic effects of reverberation in a moderate-sized classroom (5m x 9m x 3.5m). They positioned listeners at various locations: the center, left wall, back wall, and corner, and presented sounds from a range of sources in the front right quadrant. Their findings indicated that reverberation introduces variations in the interaural and spectral localization cues essential for accurate localization perception. These acoustic effects were more pronounced when the listener was near a wall and the source was far from the listener. This was explained by the fact that when the listener is far from walls, reverberation causes frequency-independent distortion. However, when the listener is close to a wall, early strong reflections lead to systematic distortions of acoustic spatial cues due to comb-filtering effects. The spectral content reaching the ears is comb-filtered, creating notches that vary with the listener’s position relative to the wall and the source’s position relative to the listener. This comb-filtering also systematically distorts interaural differences. Our findings corroborate these observations, emphasizing the significant role of dynamic acoustic cues in auditory perception. The difficulty in localizing a distant target in our study is supported by the findings of Tirado et al. [35]. For a target distance of one meter, Tirado et al. demonstrated that blindfolded participants were able to perceive the presence of a facing target effectively, but their ability to accurately locate the target was close to chance level. This illustrates that while sound reflections were detectable at that distance, localizing their source was challenging. This finding reinforces our result, especially considering that both the target used in our study and the one in Tirado et al.’s study [33] had identical dimensions. The advantage of dynamic conditions is evident in our results. When examining the impact of various acoustic conditions on the CoP-CoM postural variable (RMS) in the A/P and M/L axes, our findings showed a moderated significant effect of the acoustic condition factor on the RMS only in the A/P axis. This can be attributed to the fact that the motion of the target is in the same direction. Through multiple comparisons, significant differences were observed between the TW condition and the NE, AW, and SS conditions. The lower RMS mean values in the TW condition suggest that participants were more stable and exhibited reduced postural sway when the target was moving toward them, versus when there is no reverberant target (SS) or even when the target was static and close to them. A potential interpretation is that the dynamic acoustic cues from the approaching target in the TW condition may have facilitated better postural stabilization and balance control compared to the static target or the absence of a reflective target. The decreasing auditory distance and intensifying reflections could have provided more spatial information, enabling participants to maintain a more stable posture. Exploring specific auditory information such as echoes and their impact on the CoP-CoM as variable in identifying postural stability makes our study noteworthy in investigating the role of echoes on postural control. Most previous studies have analysed the effects of normal source sound without a reverberant target and the CoP variable. For instance, Anton et al. [36] explored the impact of various auditory stimuli on postural control in 30 healthy individuals, measuring upper body sway across different standing conditions in rooms with different reverberation times. Their findings revealed that stability increased in an echo-rich environment when interrupted auditory stimuli were presented, whereas continuous noise led to a decline in postural stability.

In a similar vein, Easton et al. [37] examined body sway in hearing-healthy subjects, observing a reduction in body sway when sound was presented through two laterally positioned loudspeakers compared to a single frontal sound source. Similarly, Ross & Balasubramaniam [38] found a decrease in the variability of body sway under exposure to white noise via headphones. Further supporting our findings, Zhong & Yost et al. [7] observed a significant reduction in body sway under auditory stimulation in both the tandem Romberg test and the Fukuda stepping test. Conversely, Maheu et al. [12] found no effect of white noise presented by a posteriorly placed sound source on stability in different standing conditions. While methodologies varied across these studies, their consistent findings reinforce our conclusion regarding the stabilizing effect of certain acoustic information on postural control. These collective results underscore the importance of auditory cues in maintaining postural stability and have guided our interest in investigating a potential link between the perception and the postural control. Unfortunately, our results did not show a significant effect of perception probability on the RMS variable, although it is worth noting that a trend was observed. This outcome may be attributed to several limitations of the study, including the small number of participants, the absence of experienced echolocators, and the inherent complexity and sensitivity of our variable.

One of the main limitations of our study lies in the fact that participants had only one familiarization trial with the auditory conditions and were not experts in echolocation. In this regard, our study differs from that of Castillo-Serrano et al. [39], who provided several hours of training (3 hours of training plus testing sessions) to examine the impact of noise on echolocation. Similarly, Norman et al. [25] investigated the effects of 20 training sessions spread over 10 weeks. Thus, although our results confirm previous findings in terms of perception, stronger conclusions could have been drawn if our participants had followed a training protocol for the auditory conditions or had been expert echolocators. Finally, we chose to use a form of “semi-active” echolocation, as the sound source was egocentric but not self-generated by the participants, unlike in active echolocation. As shown by Flanagin et al. [40], individuals achieve better perceptual results in active echolocation, when they produce the sound themselves, compared to passive echolocation. However, this requires participants to be trained in echolocation, particularly in producing mouth clicks — which was not the case in our study. Tirado [33] also highlighted the importance of training in order to practice active echolocation and produce mouth clicks. Some participants were able to localize the target using passive echolocation. However, when asked to produce their own sound, some became disoriented. This is why we opted for passive echolocation, broadcasting sound through a loudspeaker. Further research is therefore needed to examine the role of such auditory information in postural control by investigating both the effect of active echolocation using mouth clicks and the impact of echolocation training on postural parameters.”

## Conclusion

Acoustic information, particularly through the perception of echoes, appears to be involved in postural control processes alongside visual, proprioceptive, and vestibular information. However, the contribution of auditory cues to postural control seems to be relatively much lesser compared to the respective visual, proprioceptive, or vestibular systems. The stabilizing effect of auditory input on posture appears to be more pronounced when the reflective object is in motion. This suggests that dynamic auditory cues may play a more significant role in postural control compared to static auditory cues. Further research is warranted to investigate the link between auditory perception and postural control in more controlled experimental conditions. Additionally, studies involving individuals with visual impairments or blindness, who have experience with echolocation techniques, could provide valuable insights into the potential compensatory mechanisms and the extent to which auditory cues contribute to postural control in the absence of visual input. A deeper understanding of the interplay between different sensory modalities in postural control could have implications for rehabilitation strategies and assistive technologies for individuals with sensory impairments.

## Supporting information

S1 File Sound - White Noise: Synthesized white noise (frequency range of 20 Hz–20 kHz) used as stimuli. (MP3)

## References

[1] Chaudhary S, Saywell N, Taylor D. The differentiation of self-motion from external motion is a prerequisite for postural control: a narrative review of visual-vestibular interaction. Front Hum Neurosci. 2022;16:697739. doi: 10.3389/fnhum.2022.697739 35210998 PMC8860980

[2] Ivanenko Y, Gurfinkel VS. Human postural control. Front Neurosci. 2018;12:171. doi: 10.3389/fnins.2018.00171 29615859 PMC5869197

[3] Samuel AJ. A critical review on the normal postural control. POTJ. 2015;8(2):71–5. doi: 10.21088/potj.0974.5777.8215.4

[4] Massion J. Postural control system. Curr Opin Neurobiol. 1994;4(6):877–87. doi: 10.1016/0959-4388(94)90137-6 7888772

[5] Seiwerth I. Interaction of hearing and balance. Laryngorhinootologie. 2023;102(S 01):S35–49. doi: 10.1055/a-1960-4641 37130529 PMC10184668

[6] Sluydts M, De Laet C, De Coninck L, Blaivie C, van Dinther JJS, Offeciers E, et al. Case report: can cochlear implant stimulation lead to improved balance even after vestibular neurectomy?. Front Neurol. 2023;14:1248715. doi: 10.3389/fneur.2023.1248715 37693771 PMC10486889

[7] Zhong X, Yost WA. Relationship between postural stability and spatial hearing. J Am Acad Audiol. 2013;24(9):782–8. doi: 10.3766/jaaa.24.9.3 24224986

[8] Stevens MN, Barbour DL, Gronski MP, Hullar TE. Auditory contributions to maintaining balance. J Vestib Res. 2016;26(5–6):433–8. doi: 10.3233/VES-160599 28262648

[9] Ninomiya C, Hiraumi H, Yonemoto K, Sato H. Effect of hearing aids on body balance function in non-reverberant condition: a posturographic study. PLoS One. 2021;16(10):e0258590. doi: 10.1371/journal.pone.0258590 34644358 PMC8513876

[10] Shayman CS, Earhart GM, Hullar TE. Improvements in gait with hearing aids and cochlear implants. Otol Neurotol. 2017;38(4):484–6. doi: 10.1097/MAO.0000000000001360 28187057 PMC5743199

[11] Azevedo R, Teixeira N, Abade E, Carvalho A. Effects of noise on postural stability when in the standing position. Work. 2016;54(1):87–91. doi: 10.3233/WOR-162280 27061688

[12] Maheu M, Sharp A, Landry SP, Champoux F. Sensory reweighting after loss of auditory cues in healthy adults. Gait Posture. 2017;53:151–4. doi: 10.1016/j.gaitpost.2017.01.015 28157577

[13] Quijoux F, Nicolaï A, Chairi I, Bargiotas I, Ricard D, Yelnik A, et al. A review of center of pressure (COP) variables to quantify standing balance in elderly people: algorithms and open-access code. Physiol Rep. 2021;9(22):e15067. doi: 10.14814/phy2.15067 34826208 PMC8623280

[14] Winter DA, Patla A, Prince F. Stiffness control of balance during quiet standing. Gait & Posture. 1997;5(2):154–5. doi: 10.1016/s0966-6362(97)83378-4

[15] Winter DA, Patla AE, Ishac M, Gage WH. Motor mechanisms of balance during quiet standing. J Electromyogr Kinesiol. 2003;13(1):49–56. doi: 10.1016/s1050-6411(02)00085-8 12488086

[16] Winter DA, Patla AE, Rietdyk S, Ishac MG. Ankle muscle stiffness in the control of balance during quiet standing. J Neurophysiol. 2001;85(6):2630–3. doi: 10.1152/jn.2001.85.6.2630 11387407

[17] Moss CF, Ortiz ST, Wahlberg M. Adaptive echolocation behavior of bats and toothed whales in dynamic soundscapes. J Exp Biol. 2023;226(9):jeb245450. doi: 10.1242/jeb.245450 37161774 PMC10184770

[18] Centomo H, Termoz N, Savoie S, Béliveau L, Prince F. Postural control following a self-initiated reaching task in type 2 diabetic patients and age-matched controls. Gait Posture. 2007;25(4):509–14. doi: 10.1016/j.gaitpost.2006.06.010 16876995

[19] Termoz N. Le rôle des entrées auditives dans les mécanismes de régulation posturale: analyse biomécanique.

[20] Graper J. Bat/Man: echolocation, experimentation, and the question of the human. Sound Studies. 2023;10(1):59–74. doi: 10.1080/20551940.2023.2180858

[21] Thaler L, Goodale MA. Echolocation in humans: an overview. Wiley Interdiscip Rev Cogn Sci. 2016;7(6):382–93. doi: 10.1002/wcs.1408 27538733

[22] Thaler L, Castillo-Serrano J. People’s ability to detect objects using click-based echolocation: a direct comparison between mouth-clicks and clicks made by a loudspeaker. PLoS One. 2016;11(5):e0154868. doi: 10.1371/journal.pone.0154868 27135407 PMC4852930

[23] Kolarik AJ, Cirstea S, Pardhan S, Moore BCJ. A summary of research investigating echolocation abilities of blind and sighted humans. Hear Res. 2014;310:60–8. doi: 10.1016/j.heares.2014.01.010 24524865

[24] Stroffregen TA, Pittenger JB. Human echolocation as a basic form of perception and action. Ecological Psychology. 1995;7(3):181–216. doi: 10.1207/s15326969eco0703_2

[25] Norman LJ, Dodsworth C, Foresteire D, Thaler L. Human click-based echolocation: effects of blindness and age, and real-life implications in a 10-week training program. PLoS One. 2021;16(6):e0252330. doi: 10.1371/journal.pone.0252330 34077457 PMC8171922

[26] Gougoux F, Lepore F, Lassonde M, Voss P, Zatorre RJ, Belin P. Neuropsychology: pitch discrimination in the early blind. Nature. 2004;430(6997):309. doi: 10.1038/430309a 15254527

[27] Leporé N, Voss P, Lepore F, Chou Y-Y, Fortin M, Gougoux F, et al. Brain structure changes visualized in early- and late-onset blind subjects. Neuroimage. 2010;49(1):134–40. doi: 10.1016/j.neuroimage.2009.07.048 19643183 PMC2764825

[28] Thaler L, Arnott SR, Goodale MA. Neural correlates of natural human echolocation in early and late blind echolocation experts. PLoS One. 2011;6(5):e20162. doi: 10.1371/journal.pone.0020162 21633496 PMC3102086

[29] Negen J, Wen L, Thaler L, Nardini M. Bayes-like integration of a new sensory skill with vision. Sci Rep. 2018;8(1):16880. doi: 10.1038/s41598-018-35046-7 30442895 PMC6237778

[30] Read L, Deverell L. EchoRead programme: learning echolocation skills through self-paced professional development during the COVID-19 pandemic. British Journal of Visual Impairment. 2022;41(4):954–64. doi: 10.1177/02646196221131735

[31] Ashmead DH, Wall RS. Auditory perception of walls via spectral variations in the ambient sound field. J Acoust Soc Am. 2014;36(4):313–22. 10678454

[32] Prince F, Termoz N, Corriveau H, Raiche M, Roy Y, Hebert R, et al. Are 21 markers too much to define a body COM during quiet standing? 14th International Society for Posture and Gait Research.

[33] Tirado C, Lundén P, Nilsson ME. The echobot: an automated system for stimulus presentation in studies of human echolocation. PLoS One. 2019;14(10):e0223327. doi: 10.1371/journal.pone.0223327 31584971 PMC6777781

[34] Shinn-Cunningham B. Acoustics and perception of sound in everyday environments. In: Proceedings of the 3rd Int. Workshop on Spatial Media.

[35] Tirado C, Gerdfeldter B, Kärnekull SC, Nilsson ME. Comparing echo-detection and echo-localization in sighted individuals. Perception. 2021;50(4):308–27. doi: 10.1177/03010066211000617 33673742 PMC8044610

[36] Anton K, Ernst A, Basta D. Auditory influence on postural control during stance tasks in different acoustic conditions. J Vestib Res. 2019;29(6):287–94. doi: 10.3233/VES-190674 31450523

[37] Easton RD, Greene AJ, DiZio P, Lackner JR. Auditory cues for orientation and postural control in sighted and congenitally blind people. Exp Brain Res. 1998;118(4):541–50. doi: 10.1007/s002210050310 9504849

[38] Ross JM, Balasubramaniam R. Auditory white noise reduces postural fluctuations even in the absence of vision. Exp Brain Res. 2015;233(8):2357–63. doi: 10.1007/s00221-015-4304-y 25953650

[39] Castillo-Serrano JG, Norman LJ, Foresteire D, Thaler L. Increased emission intensity can compensate for the presence of noise in human click-based echolocation. Sci Rep. 2021;11(1):1750. doi: 10.1038/s41598-021-81220-9 33462283 PMC7813859

[40] Flanagin VL, Schörnich S, Schranner M, Hummel N, Wallmeier L, Wahlberg M, et al. Human exploration of enclosed spaces through echolocation. J Neurosci. 2017;37(6):1614–27. doi: 10.1523/JNEUROSCI.1566-12.2016 28073936 PMC6705675

